# Somatostatin receptor expression, tumour response and quality of life in patients with advanced hepatocellular carcinoma treated with long-acting octreotide

**DOI:** 10.1038/sj.bjc.6603674

**Published:** 2007-04-03

**Authors:** J Cebon, M Findlay, C Hargreaves, M Stockler, P Thompson, M Boyer, S Roberts, A Poon, A M Scott, V Kalff, G Garas, A Dowling, D Crawford, J Ring, R Basser, A Strickland, G Macdonald, M Green, A Nowak, B Dickman, H Dhillon, V Gebski, Australasian Gastro-Intestinal Trials Group (AGITG) AG0001H Investigators

**Correction to:** *British Journal of Cancer* (2006) **95**, 853–861. doi: 10.1038/6603325

Owing to an error on the author's part, the full list of authors was not given when the paper was originally published. The authors would like the authorship of the paper to be properly reflected; the full, accurate list of authors is therefore listed, above.

